# Interactions between Population Density of the Colorado Potato Beetle, *Leptinotarsa decemlineata*, and Herbicide Rate for Suppression of Solanaceous Weeds

**DOI:** 10.1673/031.008.3801

**Published:** 2008-05-14

**Authors:** Chase Metzger, Rick Boydston, Holly Ferguson, Martin M. Williams, Richard Zack, Doug Walsh

**Affiliations:** ^1^ Washington State University Long Beach Research and Extension Unit, 2907 Pioneer Rd, Long Beach, WA 98631; ^2^ USDA-ARS, Irrigated Agriculture Research and Extension Center, 24106 North Bunn Road, Prosser, WA 99350; ^3^ Washington State University, Irrigated Agriculture Research and Extension Center, 24106 North Bunn Road, Prosser, WA, 99350; ^4^ USDA-ARS, 1102 S. Goodwin Avenue, University of Illinois, Urbana, IL 61801; ^5^ Department of Entomology, Washington State University, Pullman, WA 99164

**Keywords:** *Solanum tuberosum*, *Solanum physalifolium*, *Solanum triflorum*, cutleaf nightshade, hairy nightshade

## Abstract

The presence of volunteer potato *Solanum tuberosum* L., cutleaf nightshade, *S. triflorum* N., and hairy nightshade, *S. physalifolium* Rusby (Solanales: Solanaceae), throughout potato crop rotations can diminish the effectiveness of crop rotations designed to control disease and pest problems associated with growing potatoes. In greenhouse bioassays, larvae of the Colorado potato beetle, *Leptinotarsa decemlineata* Say (Coleoptera: Chrysomelidae) were placed in population densities of 0, 5, 10, and 40 per potato (cv. Russet Burbank) plant and 0, 5, 10, and 15 per cutleaf nightshade and hairy nightshade plant. Plants were treated with different rates of herbicides including fluroxypyr, prometryn, and mesotrione rates, and the physiological response on the potato plants was assessed by weighing shoot biomass 14 days after treatment. Consistently, across all bioassays, rate response functions were shifted as *L. decemlineata* density increased, such that less herbicide was required to achieve control. For instance, the herbicide rate needed to achieve 90% reduction in potato biomass was reduced from 62 to 0 g fluroxypyr per hectare and 711 to 0 g prometryn per hectare as *L. decemlineata* density was increased to 40 larvae per plant. Herbivory at higher *L. decemlineata* population densities and herbicides above certain rates resulted in large reductions in cutleaf and hairy nightshade biomass. Differences in rate response functions among *L. decemlineata* population densities indicated that *L. decemlineata* contributed to weed suppression in combination with herbicides. These data suggest that integrated weed management systems targeting volunteer potato, cutleaf nightshade, and hairy nightshade can be more effective when herbicide applications are combined with herbivory by naturally occurring Colorado potato beetles.

## Introduction

Potatoes, *Solanum tuberosum* L. (Solanales: Solanaceae), an introduced species are commonly grown in the northwestern United States on a 3- to 4-year rotation with other crops such as peas, spring wheat, mint, corn, and carrots in order to break disease and pest cycles associated with potatoes. However, the persistence of solanaceous weed species and presence of volunteer potatoes throughout the rotation can greatly reduce the benefits of such disruptions (Williams and Boydston 2002; Boydston et al., 2007). Lutman (1977) found that potato tuber population density after harvest could exceed the population densities used for commercial plantings, with as many as 370,000 tubers per hectare remaining and up to 50,000 sprouts per hectare recorded in winter wheat fields. In Washington State, studies have shown that as many as 450,000 tubers per hectare remain in the soil following potato harvest (Steiner et al. 2005).

The Colorado potato beetle (*Leptinotarsa decemlineata* Say) (Coleoptera: Chrysomelidae) is a common defoliating pest of solanaceous crops and may be able to suppress volunteer potatoes and other solanaceous weeds such as nightshades in potato crop rotations. *L. decemlineata* feeds on solanaceous plants including potato and two common weeds in potato growing regions: hairy nightshade (*S. physalifolium* Rusby, formerly *S. sarrachoides* S. (Miller and Parker 2006)) and cutleaf nightshade (*S. triflorum* N.). Several studies have demonstrated that nightshade species are more suitable host plants than potato for *L. decemlineata* (Horton and Capinera 1990; Xu and Long 1995, 1997). *L. decemlineata* has two generations per year in the Columbia Basin of the Pacific Northwest with adults emerging from diapause in late April to early May (Berry 1998; Xu and Long 1997). Thus adults are present and feeding at the time when volunteer potato and other solanaceous weeds are emerging. Adult females lay eggs in masses of 10 to 30 (Boiteau and LeBlanc 1992; Berry 1998). *L. decemlineata* is a spatially aggregated species, the first aggregations arising from the egg hatches (Boiteau 2005). Larvae undergo four instars; the fourth instar burrows into the soil and pupates. Fourth instar larvae and adults are the life stages that consume the most plant matter (Ferro et al. 1985). Second generation *L. decemlineata* adults emerge, feed, and then enter diapause (Berry 1998) that is triggered by shortening day length and deteriorating host plant quality (Boiteau and LeBlanc 1992). Adults overwinter in the soil (Lefevere et al. 1989); some may even enter a prolonged diapause of 2 to 4 years (Biever and Chauvin 1990), thus allowing them to remain in the field throughout the crop rotation cycle. Beetles typically walk into the field from overwintering sites surrounding the field, creating an edge effect (French et al. 1993; Boiteau 2001), though tubers and volunteer potato present throughout the rotation create potential overwintering sites within the field.

Miller and Parker (2006) listed hairy nightshade, black nightshade (*S. nigrum*), and cutleaf nightshade as the three predominant annual nightshade species found in cropping systems in the Pacific Northwest region of the United States, with hairy nightshade being the most common. Hairy nightshade is an introduced species from South America (Miller and Parker 2006). Cutleaf nightshade is native to Washington east of the Cascade Mountains (Whitson et al. 2006) and black nightshade is native to Eurasia (Ogg and Rogers 1989). Most nightshade seedlings appear in late April in the Pacific Northwest and continue emerging through June. Little germination occurs in July, but late-emerging seedlings appear in August and these weeds can contaminate the crop harvest (Miller and Parker 2006).

Xu and Long (1997) observed that volunteer potato was the first food source for overwintered adult *L. decemlineata* in late April/early May in the Columbia Basin of Washington State. *L. decemlineata* adults were detected slightly later on cultivated potato that emerged later than volunteer potato in the rotated crop. Their mark-recapture studies suggested that first generation adults that developed on volunteer potato could potentially disperse to nightshade weeds when volunteer potato vines senesced as the crop canopy closed. The presence of defoliating *L. decemlineata* in coincidence with the beetles' preferred host plants in a cropping system suggests that naturally occurring *L. decemlineata* herbivory could be used in an integrated weed management program against volunteer potato and nightshade species at no cost to the grower (Williams et al. 2004; Boydston and Williams 2005).

Volunteer potato is difficult to control because it is able to sprout numerous times from the mother tuber following shoot removal via herbicide use or cultivation (Williams and Boydston 2002). Integrated control efforts involving herbicide and/or other management tactics are often not effective (Williams et al. 2004). Similarly, the three common nightshade species have shown tolerance to commonly used herbicides such as pendimethalin, trifluralin, and metribuzin (Miller and Parker 2006). Fluroxypyr ([(4-amino-3,5-dichloro-6-fluoro-2-pyridinyl)oxy] acetic acid) an auxin inhibitor, prometryn (*N,N*-bis (1-methylethyl)-6-(methylthio)-1,3,5-triazine-2,4-diamine) a photosystem II inhibitor, and mesotrione (2-[4-methylsulfonyl)-2-nitrobenzoyl]-1,3-cyclohexanedione) a carotenoid synthesis inhibitor, are three herbicides reported to have good efficacy against volunteer potatoes and nightshade species in several of the crops rotated with potato (Williams et al. 2004; Boydston and Williams 2005; Williams and Boydston 2005). However, there are certain circumstances in which the effectiveness of these herbicides could be improved with the integration of *L. decemlineata* herbivory. Incomplete control of larger weeds or lack of control of later emerging weeds could be improved with *L. decemlineata* herbivory. In addition, herbicide control of solanaceous weeds taller than the crop is not cost-effective and may even result in further crop destruction. Combining beetle feeding with a field rate of herbicide in this situation would not add to the control costs and could potentially reduce the weed fitness sufficiently to allow the crop to recover.

Previous work (Williams et al. 2004) demonstrated in greenhouse and field bioassays that fluroxypyr in the absence of *L. decemlineata* larval herbivory reduced potato leaf area and shoot biomass by up to 88% and 69%, respectively. The I50 rate (rate eliciting a 50% response) of fluroxypyr for leaf area and shoot biomass without *L. decemlineata* feeding was 8 and 9 g ae/ha, respectively. When they added *L. decemlineata* herbivory, the I50 rate was lowered to 3 g ae/ha. These results suggest that combining fluroxypyr with *L. decemlineata* herbivory may allow for weed suppression with lower rates of fluroxypyr. Using effective herbicides at full rates for volunteer potato suppression in field corn, Boydston and Williams (2005) reported that *L. decemlineata* herbivory contributed little to overall suppression of volunteer potato. However, where herbicides were not used, *L. decemlineata* herbivory reduced tuber number and tuber mass by 21% and 23%, respectively. In the field experiments of both of these studies, *L. decemlineata* density was not controlled in the herbivory-present plants, which resulted in highly variable defoliation in those treatments. Although large reductions in leaf area were observed for all herbicide plus herbivory treatments, except fluroxypyr, by Boydston and Williams (2005), these differences were not significant. To address the high variability issue, Williams et al. (2004) suggested further research was needed involving specific beetle density levels in combination with herbicide treatments.

The objective of this study was to expand on the work done by Williams et al. (2004) by testing different density levels of *L. decemlineata* in combination with a range of rates of fluroxypyr, prometryn, and mesotrione on volunteer potato as well as hairy and cutleaf nightshade all of which serve as host plants for *L. decemlineata* in the same cropping systems. In addition, experiments were conducted under controlled conditions in the greenhouse to enable us to better analyze the effects of herbicide rate and *L. decemlineata* larval herbivory as well as their interactions on solanaceous weed suppression.

## Materials and Methods

### Plant material and insects

Potatoes (cv. Russet Burbank) were grown from commercially grown tubers harvested the previous fall and stored at 4.4°C until planting. Potatoes were grown using 5 g single tuber eyes planted 2.5 cm deep in 2 L pots in all experiments, except the prometryn bioassay, in which 7.6 L pots were used. Cutleaf and hairy nightshade were grown, using field-collected seed, in 2 L pots. They were fertilized with one application of 15 ml granular 5-3-1 Gro-Power™. Pots were sub-irrigated by filling a plastic dish that contained the pot. *L. decemlineata* egg masses were obtained from a greenhouse-reared population and from fields throughout the Columbia Basin of Washington and were incubated in plastic petri dishes at room temperature until hatch.

### Greenhouse conditions

All bioassays were conducted in greenhouse conditions. The greenhouse temperature was monitored using a HOBO (www.onsetcomp.com) Pro Series Temp, RH monitor with a daily average of 25.6 ± 4.9 °C and 16:8 L:D.

### Herbicides and *L. decemlineata* population densities

**Figure 1.**
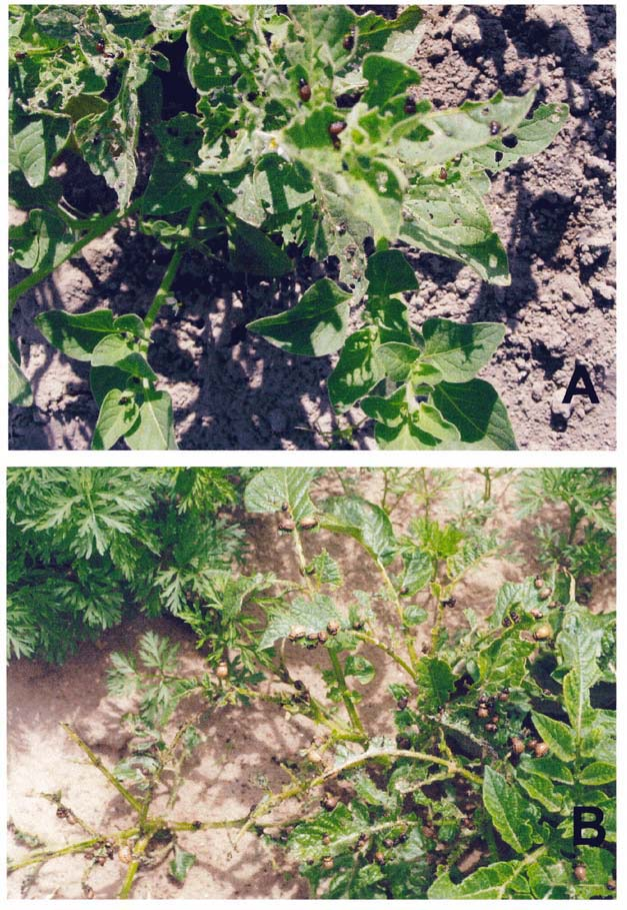
Colorado potato beetle larvae feeding on hairy nightshade in onions (A) and on volunteer potato in carrots (B) during midseason in southeastern Washington State.

Potatoes were treated with six rates of fluroxypyr and prometryn, cutleaf nightshade was treated with four rates of mesotrione, and hairy nightshade was treated with four rates of fluroxypyr. Potato plants were subjected to *L. decemlineata* population densities of 0, 5, 10, and 40 larvae per plant. Cutleaf and hairy nightshade were subjected to population densities of 0, 5, 10, and 15 larvae per plant. The density levels chosen were based primarily on *L. decemlineata* oviposition behavior as eggs are laid in masses of 10 to 30 or more eggs (Berry 1998) with a lower density level added to determine the lowest number of beetles needed to achieve an effect on the weeds. Frequently, in the field, as many as 15 to 20 larvae may be found on a single plant (Figure 1) but not on every plant. The mean densities per plant reported in the literature are generally less than 10 per potato plant (Xu and Long 1997; Williams et al. 2004; Boydston and Williams 2005) though mean densities exceeding 85 larvae per hairy nightshade plant have been recorded (Xu and Long 1997). For the nightshade experiments, the highest density level was much lower than for potato because there were fewer beetle larvae available for these experiments. Treatments were replicated four times. *L. decemlineata* mortality was monitored and the population replenished with live larvae to maintain the desired population density. New potato shoots arising from the seed tuber were removed to limit between-plant variations in shoot number.

Herbicides were applied using a bench sprayer with a single even flat fan nozzle (Teejet 80015E, www.teejet.com) that delivers 233 L per hectare at 193 kPa moving 1.61 km per hour. Potatoes were subjected to the herbicide and beetle treatments when they were 18.3 ± 3.3 cm tall and had four to six leaves. This occurred approximately one month after planting, 20 days after shoot emergence. Herbicides were applied to the nightshade species at flowering, approximately two months after shoot emergence.

Plants were harvested 14 days after herbicide application. Plants were clipped at the soil surface, and the stems and leaves (shoot biomass) were oven-dried at 70°C for 48 hours and weighed.

Four separate, replicated experiments are described in the next four sections. Beetle herbivory conditions varied with each experiment, depending on the availability of beetle larvae as well as host plants at the same developmental stage.

### Fluroxypyr bioassay with potato

Fluroxypyr was applied at 0, 14, 28, 56, 112, 224 g ae per hectare. *L. decemlineata* first instar larvae less than 48 hours old were transferred onto potato foliage within 3 hours of herbicide application. Dead larvae were replaced for up to 8 days after herbicide application to maintain the desired level of herbivory. Larvae were replaced only to 30 per plant for the highest population density of 40 larvae per plant, due to the rapid defoliation of plants. Larvae were allowed to feed until available forage was depleted, at which time remaining larvae in the high population density of 40 larvae per plant were transferred onto plants with available forage in the lower population densities of 5 and 10, to maintain the population density on those plants after some larvae had pupated. Pupation usually occurred 8 days post-spray on all treatments.

### Prometryn bioassay with potato

Prometryn was applied to potatoes at 0, 280, 560, 1121, 2242, and 4484 g ai per hectare. *L. decemlineata* first instar larvae less than 48 hours old were transferred onto potato foliage within 72 hours of herbicide application with the high population density of 40 larvae reached between 0 and 7 days. Dead larvae were replaced for up to 12 days after herbicide application to maintain the desired population density on plants not completely defoliated.

Replications one and two received 2 hours of artificial light, which supplemented natural daylight for 11 and 7 days post-spray, respectively. However, the plants were not exhibiting characteristic herbicide response. Subsequently, the amount of artificial light was changed from 2 hours to 8.5 hours for replications three and four, which resulted in characteristic plant response to the prometryn in all replications.

### Mesotrione on cutleaf nightshade

Mesotrione was applied on cutleaf nightshade at 0, 6.6, 26.4, and 105 g ai per hectare. All four replications were sprayed on the same day and *L. decemlineata* larvae were transferred immediately to plants. After 8 days post-spray, larvae were removed from plants that were completely defoliated and redistributed to plants in which population densities had fallen below the initial intended population density.

### Fluroxypyr on hairy nightshade

Fluroxypyr was applied on hairy nightshade at 0, 14, 56, and 224 g ae/ha. *L. decemlineata* larvae less than 48 hours old were imposed on each plant within 3 hours of herbicide application. Replications one and two were sprayed three days apart, and replications three and four were sprayed simultaneously one month later.

### Experimental design

The experimental design was a randomized complete block with four replications. Each plant was one experimental unit. The potato studies were arranged in a 6 × 4 factorial arrangement, while the nightshade studies were arranged in a 4 × 4 factorial arrangement, with factors of herbicide rate and *L. decemlineata* population density, respectively.

### Statistical analyses

Data were analyzed separately for each of the four experiments. One-way ANOVA (α = 0.05) was used to determine if there was a significant replicate effect on shoot biomass. The data were then pooled to test for the effects of herbicide rate and *L. decemlineata* population density on shoot biomass using a two-way ANOVA (α = 0.05). Herbicide rate and *L. decemlineata* population density effects were further analyzed using one-way ANOVA and Fisher's protected LSD test (α = 0.05). These analyses were performed with Minitab 14 statistical software.

A logistic model was used to quantify weed biomass over a range of herbicide rates (Seefeldt et al. 1995): 
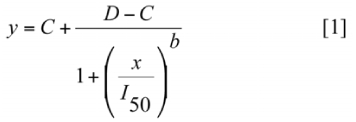
where, *y* = weed shoot biomass, *x* = herbicide rate, *C* = biomass at high rates (lower asymptote), *D* = shoot biomass when rate equals 0 (upper asymptote), *I50* = rate eliciting 50% reduction in shoot biomass, and *b* = the slope at the *I_50_* rate. Within each trial, Equation 1 was fit to weed shoot biomass for each *L. decemlineata* population density using an iterative least squares procedure (Systat 11, www.systat.com). Lack of fit was assessed by reporting standard errors of parameter estimates, calculating *R*2 values, and reporting root mean square errors. The extra sum of squares principle for nonlinear regression analysis was employed to evaluate the similarity of parameter estimates for *L. decemlineata* population densities (Ratkowsky 1983). Comparisons were made by calculating a variance ratio of individual and pooled residual sums of squares and performing an *F* test at α = 0.05.

Once model parameters were determined, an additional parameter was calculated. This parameter was aimed at quantifying the herbicide rate, under herbivory, required to reduce weed biomass to a value equivalent to a 90% reduction without herbivory. A fixed response rate *(FRR)* serves as a comparison between two rate response functions (e.g., with and without herbivory) at a fixed plant response (Williams et al. 2004). Model parameters from Equation 1, in the absence of herbivory, were used to calculate a 90% reduction (*ϒ_90_*) in weed biomass, relative to herbicide rate = 0. The equation used to solve for *ϒ_90_* was: 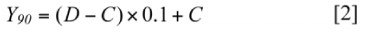
in which *D* and *C* are the same parameters in Equation 1. The *FRR* was determined by rewriting Equation 1, using the *ϒ_90_* parameter from Equation 2, and solving for rate:

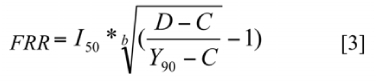

with parameters described above. An *FRR* was calculated for each level of herbivory using parameter estimates from Equations 1 and 2.

**Figure 2.**
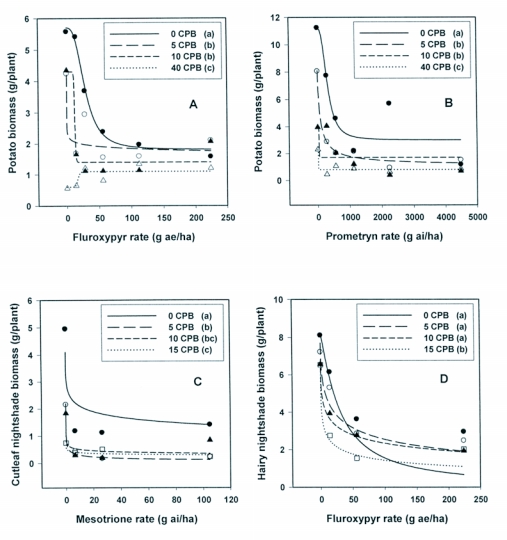
Weed biomass as a function of herbicide rate and Colorado potato beetle (*L. decemlineata*) herbivory in four greenhouse trials. Herbivory level is the number of larvae maintained on plants for the duration of the trials and mean observations are identified as 0 larvae in filled circles, 5 larvae in open circles, 10 larvae in filled triangles, 15 larvae in open squares (C and D only), 40 larvae in open triangles (A and B only). Equation I was fit to all observations within each *L. decemlineata* herbivory level and parameter estimates are reported in Table 2. Within each trial, similarity in dose response functions among herbivory levels was tested using the extra sum of squares principle for nonlinear regression. Herbivory levels followed by the same letter in parentheses are similar (*F* - test, α = 0.05).

## Results and Discussion

### Fluroxypyr bioassay with potato

Potato shoot biomass was significantly (*P* <0.01) reduced with increasing fluroxypyr rate or *L. decemlineata* population density, with a highly significant (*P* <0.01) interaction effect between fluroxypyr rate and population density (Table 1). Herbicide rate response was influenced by population density. Potato shoot biomass was reduced by all larval densities and fluroxypyr rates. A 69% reduction in shoot biomass was observed with the lowest rate of fluroxypyr in combination with the lowest density levels of 5 and 10 larvae per plant (Figure 2A). Fitting these data to a logistic model and calculating the *ϒ_90_* for shoot biomass and the FRR allow quantification of the least amount of herbicide required to result in a practical outcome for different population densities. To obtain a 90% reduction in shoot biomass, 62 g ae/ha fluroxypyr were required when larvae were absent, whereas 3, 13, and 0 g ae/ha fluroxypyr were sufficient with population densities of 5, 10, and 40, respectively (Table 2). Herbivory from 40 larvae per plant resulted in plant death regardless of fluroxypyr rate. These results are consistent with Williams et al. (2004) who reported that the biologically effective fluroxypyr rate for 95% reduction in volunteer potato shoot biomass was 41 g ae/ha in the absence of herbivory, compared with 18 g ae/ha when 20 larvae per plant fed for 8 to 11 days.

### Prometryn bioassay with potato

Potato shoot biomass was significantly (*P* <0.01) reduced with increasing prometryn rate or *L. decemlineata* population density with a highly significant (*P* <0.01) interaction (Table 1). Similar to the fluroxypyr bioassay, potato response to herbicide was influenced by *L. decemlineata* density. Biomass reductions occurred sooner when beetle herbivory was combined with herbicide, even at the lowest beetle density level and prometryn rate (Figure 2B). While rate response functions were similar between 5 and 10 larvae per plant, additional reduction in shoot biomass was observed in the plants subjected to 40 larvae per plant at all prometryn rates. As an example, the *I*_50_ rate for potato shoot biomass in the absence of herbivory was 315 g ai/ha compared with 84, 40, and 15 g ai/ha in population densities of 5, 10, and 40, respectively. The ability of increased herbivory to reduce herbicide required is further evidenced by the fixed response rate. To obtain a 90% reduction in shoot biomass, 711 g ae/ha was required when *L. decemlineata* were absent, whereas 139, 31, and 0 g ae/ha were sufficient with population densities of 5, 10, and 40 per plant, respectively (Table 2).

**Table 1.**
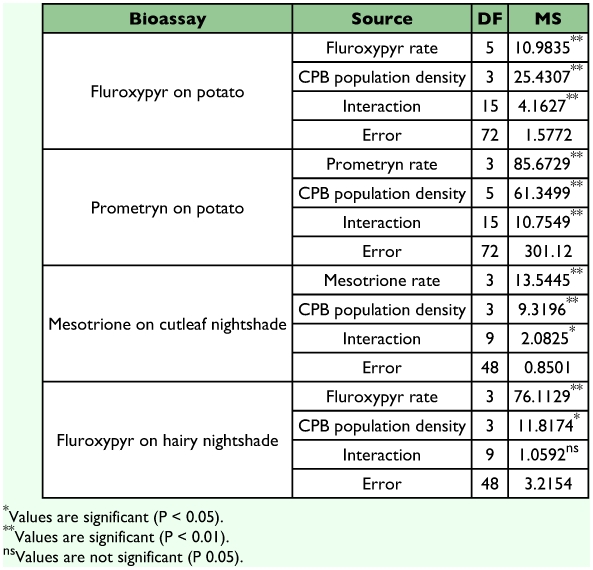
Analysis of variance results (mean square values) for the effect of CPB population density × herbicide rate on shoot biomass.

### Mesotrione bioassay with cutleaf nightshade

Cutleaf nightshade shoot biomass per plant was significantly reduced (*P* <0.01) by mesotrione rate or *L. decemlineata* population density with a significant (*P* <0.05) interaction (Table 1). Population densities also influenced response of cutleaf nightshade to mesotrione rate. The *D* parameter (upper asymptote) of Equation 1 decreased from 4.1, 2.2, 1.8, and 0.8 g per plant as larval density increased from 0, 5, 10, to 15 larvae per plant, respectively (Table 2). Defoliation was rapid in the presence of larvae, and differences among population density treatments at most individual rates or response levels were not apparent. Nonetheless, herbivory influenced herbicide rate response as confirmed by the *F*-test comparing rate response functions among population densities (Figure 2C). Feeding injury caused by 15 larvae per plant was significantly different from injury caused by 5 larvae per plant. Although data on lower rates of mesotrione used in combination with beetle herbivory may further elucidate their interactive effects toward the upper asymptote of the rate response functions, those rates may not be of practical application in the field.

### Fluroxypyr bioassay with hairy nightshade

Hairy nightshade shoot biomass was reduced by fluroxypyr and larval herbivory; however no significant (*P* ≥ 0.05) interaction occurred (Table 1). Herbivory alone reduced hairy nightshade biomass by 21% as larval density increased to 15 larvae per plant, a relatively subtle difference compared with plant response in other bioassays. Likewise, few significant differences were observed among rate response functions for larval density levels (Figure 2D). However, it should be noted that the rate response functions for herbivory plus herbicide for the three larval density levels showed more rapid reduction of plant biomass as herbicide rate increased compared with the function for herbicide treatment alone (Figure 2D). Moreover, there was a trend for the *I_50_* rate to decline from 30 to 4.2 g ae/ha as larval density increased (Table 2). Practical application of these data toward determining a biologically effective herbicide rate was difficult since the fixed response rate *(FRR)* of most *L. decemlineata* population densities exceeded the maximum rate used in the study. Though data on lower rates of fluroxypyr used in combination with beetle herbivory on hairy nightshade may further elucidate their interactive effects toward the upper asymptote of the rate response functions, those rates may not be of practical field application.

**Table 2.**
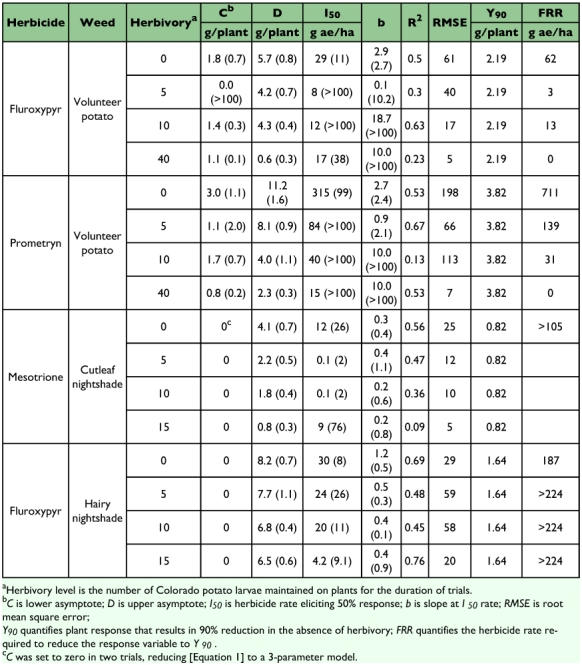
Parameter estimates [Equation 1] for the effects of herbicide rate and *Leptinotarsa decemlineata* population density on weed shoot biomass (standard errors in parentheses).

The results presented here suggest that integrating herbivory from naturally occurring *L. decemlineata* with herbicides such as fluroxypyr, prometryn, or mesotrione in potato crop rotations may complement the efficacy of those herbicides on volunteer potato, hairy nightshade, and cutleaf nightshade. Companion field studies with different beetle density levels and herbicide rates should be done to test these results in the field and determine the practical applications of combining beetle herbivory with reduced herbicide rate for less expensive, but effective solanaceous weed control. Field-related factors should be taken into consideration, such as competition and shading from the crop, which may influence the optimum rates of *L. decemlineata* density and herbicide needed for weed control.

To best utilize naturally occurring beetle herbivory in an integrated weed management system, *L. decemlineata* population abundance as well as weed developmental stage and abundance would need to be monitored regularly throughout the season from the time of weed or volunteer potato emergence. In addition, optimized timing of herbicide application is essential to get the most benefit from the addition of beetle herbivory. Based on sampling conducted in 2005 in peppermint, spring wheat, organic sweet corn, and field corn, *L. decemlineata* populations did not reach sufficient levels to defoliate potatoes adequately and allow a reduction in herbicide rate for control of volunteer potato until well after tuber set (Metzger 2005). Boydston and Seymour (2002) observed that *L. decemlineata* severely defoliated volunteer potato not treated with herbicide in an onion crop in late July and August, but this was too late to prevent onion yield loss from early-season weed competition. These observations suggest the need for early-season herbicide use to suppress volunteer potato development until sufficient *L. decemlineata* populations of the second generation have built up. Data presented here suggest that an effective target beetle density for volunteer potato suppression would be 5 to 10 beetles or more per plant when used in combination with fluroxypyr or prometryn. For cutleaf nightshade, a minimum of 5 beetles per weed plant in combination with a low rate of mesotrione is suggested, but for hairy nightshade, a higher minimum of 15 beetles per plant in combination with a rate of up to 56 g ae/acre fluroxypyr may be needed.

When considering the use of beetle herbivory in a weed control program, it is important to assess the distribution of *L. decemlineata* within the field. If the beetles were present in the previous crop of potatoes, they could overwinter in the soil within the field and be present throughout the field wherever volunteer potato emerges in the next crop. However, fields that lack overwintering *L. decemlineata* would more than likely have immigrating postdiapause beetles at the field edges followed by dispersal of the beetles throughout the field to weed host plants. This was found in potato fields where postdiapausing adults in the spring were shown to initially aggregate at the field borders and soon after disperse throughout the remainder of the field within one week of arrival at the field edge (Boiteau 2005).

The extent to which unchecked *L. decemlineata* populations would influence *L. decemlineata* management in the following potato crop merits careful consideration. Broad-spectrum insecticides targeting pests in the crop rotated after potato would also reduce *L. decemlineata* populations. However, allowing even a low population density of *L. decemlineata* to defoliate the weeds before or after an herbicide application may reduce herbicide amounts needed to achieve acceptable weed suppression and may lower pesticide costs for the grower.

## **Abbreviations:**

150 -: rate eliciting 50% reduction in shoot biomass
Y90 -: 90% reduction in weed biomass, relative to herbicide rate = 0
